# Supplementary material for: Resource partitioning among brachiopods and bivalves at ancient hydrocarbon seeps: A hypothesis
Source: PLoS One. 2019 Sep 5;14(9):e0221887. doi: 10.1371/journal.pone.0221887 (PMC6728048; doi:10.1371/journal.pone.0221887)
Supplement: Attachment — Submitted filename: Kiel_Peckmann_2019_review.docx [file pone.0221887.s001.docx]

This is a review of manuscript „Resource partitioning among brachiopods and bivalves at ancient hydrocarbon seeps: a hypothesis” by Steffen Kiel and Joern Peckmann. The manuscript tackles and interesting topic of seemingly contrasting brachiopod and bivalve evolutionary histories at seeps throughout the Phanerozoic. The authors present an interesting hypothesis that bivalves and brachiopods utilized different resources, and that the contrasting evolutionary pathways are apparent rather than real. Authors present evidence to support this hypothesis, which makes the manuscript a quality work eligible for publication after a minor revision.

I have reviewed the previous version of this manuscript, where I have pointed several drawbacks in the reasoning, which were improved since then. The manuscript is properly referenced, although there are several important references missing, as I outline below. There are some minor drawbacks left to be corrected, outlined below.:

Line 40: please don’t get overly enthusiastic, it is difficult to judge what is most, and what is least extreme environment, it all depends on for whom; “…a similar pattern was also seen at deep-sea hydrothermal vents and hydrocarbon seeps” is enough

Line 182: we are unable to say whether they indeed have never developed methanotrophic symbioses, or they developed them only to have those symbioses extinct; better to write: Remarkable in this context is that other bivalve families with intracellular symbionts have not developed methanotrophic symbioses, or have not developed methanotrophic symbioses that would survive to the present day

Line 261-263: *Phreagena kilmeri*, rather than *Archivesica kilmeri* (check out vesicomyid taxonomy update paper by Krylova and Sahling (2010), PLoS one; line 262: *C*. *pacifica*, rather than *P*. *pacifica*

Line 308-309: Remove the sentence “Despite the presence of early diagenetic fibrous cement, *Anarhynchia smithi* probably lived in a low sulfide environment.” It doesn’t bring anything into the discussion, later you give some reasons why this might have been a low sulfide seep.

Line 332-333: There are two Greylock Butte seep deposits; one contains micrite, mass accumulations of *Halorella* up to 10 cm along longer axis, and no other fossils; the second Greylock Butte deposit contains somewhat rare *Halorella* up to 3-4 cm along longer axis, and some molluscs (anomalodesmatan and modiomorphid bivalves, among others); it is not true that both seep deposits in Oregon contain mass accumulations of *Halorella*, both are different, with second one being somewhat similar to Turkish deposit; please rewrite accordingly.

Line 350-351: remove “which, in turn, was suitable for the filter-feeding brachiopods”. Unnecessary.

Line 385-386: *Eucalathis methanophila* at Omagari occurs in some sort of monospecific brachiopod accumulations, although it doesn’t for such mass accumulations as dimerelloids did, please consider that; please add citation of Hryniewicz et al. (2019), describing another non-dimerelloid brachiopod from seep, *Neoliothyrina nakremi*

Another figure, with two schematic subfigures illustrating geochemical gradients and fauna at low-sulfide seeps with dimerelloids, and high sulfide seeps with bivalves, respectively, would be useful in this manuscript.

Table 1: clarify why you asterisk fossil genera erected after 1995 (presumably because after Campbell and Bottjer paper); you should asterisk also extant genera with fossil record erected after 1995 (they were unknown for Campbell and Bottjer, just as the fossil genera were)

Neogene:

*Cubatea*, rather than *Cubathea*, consider asterisking *Elliptiolucina*, *Meganodontia* according to my previous comments

*Adulomya* vs. *Pleurophopsis*: in your (SK) recent paper on Japanese Neogene vesis you consider *Adulomya* and *Pleurophopsis* as synonyms, with *Pleurophopsis* having priority. These seems to be true at least for Neogene species. Please be consistent here.

*Samiolus* from your 2017 paper (SK) paper on Miocene chemosymbiotic seep bivalves from Italy is missing – why? Also “*Anodontia*” from Ca’Fornace is missing (citation: Kiel et al. 2018), and *Megaxinus* from Stirone river (citation: Kiel and Taviani 2018), please complement the reference list and adjust fig. 1 according to missing genera

Paleogene:

*Cubatea*, not *Cubathea*

add *Rhacothyas* (citation: Hryniewicz et al. 2019), also *Solemya* species recorded there; adjust figure 1

*Adulomya*/*Pleurophopsis* problem again

Jurassic: Please note that there were 15 seep carbonates in Sassenfjorden area, with 3 of them Jurassic in age and the 8 Cretaceous (remainder not dated; details of stratigraphy in Wierzbowski et al. 2011, available from me on request). Therefore, putting all Sassenfjorden seep carbonates into Early Cretaceous category is an error. The significance of that is *Solemya* from one of the Jurassic seeps from Sassenfjorden, which is missing from your list and should be added (cite Hryniewicz *et al*. 2014, Zootaxa)

Table 2

be consistent and use “Fm” or “fm”; some of the terms used later are not lithostratigraphic in any way, but geographic (e.g. Beskidy Range, Musenalp, Wollaston Forland), therefore

Neogene: Ca’Fornace seep deposit missing (citation: Kiel *et al*. 2018)

Paleogene

Paleocene seep sites from Spitsbergen from Spitsbergen missing (citation: Hryniewicz *et al*. 2016, Palaeo3x, describing site, not fauna; do not cite 2019 paper here as it discusses the fauna)

Satsop Weatherwax seep deposit missing (citation: Hybertsen and Kiel, 2018, APP)

Late Cretaceous: separate Omagari lens and Yasukawa seep, these are two very different deposits

There are two Late Cretaceous seep deposits on Antarctic Peninsula; one on Seymour Island (Lopez de Bertodano Fm), one on Snow Hill Island (Snow Hill Island Formation), both are lumped into one here which is a mistake; worse, the deposit from Seymour Island is referred to as belonging to Snow Hill Island Formation, the seep-bearing lithologies of which do not even cropp out on Seymour Island; please correct that

Devonian

Regarding Sidi-Amar: in your 2007 paper you write about a limestone layer yielding *Dzieduszyckia* which was sampled, please include that next to “erratic limestone”; and “Devonian erratic limestones” does not look good as it suggest glacial erratics to most people, perhaps write “Devono-Carboniferous melange” or something like that

Despite numerous studies, I have seen the host lithological unit of Hollard Mound named just once, and that would be *Pinacites* limestone indeed (Peckmann et al. 1999); this is an informal unit, so small “l” and italics for *Pinacites*

Missing references:

Hryniewicz, K., Little, C.T.S., and Nakrem, H.A. 2014. Bivalves from the latest Jurassic–earliest Cretaceous hydrocarbon seep carbonates from Spitsbergen, Svalbard. Zootaxa 3859: 1–66.

Hryniewicz, K., Bitner, M.A., Durska, E., Hagström, J., Hjálmársdottir H.R., Jenkins, R.G., Little, C.T.S., Miyajima, Y., Nakrem, H.A., and Kaim, A. 2016. Paleocene methane seep and wood-fall marine environments from Spitsbergen, Svalbard. Palaeogeography, Palaeoclimatology,Palaeoecology 462: 41–56.

Hryniewicz, K., Amano, A., Bitner, M.A., Hagström, J., Kiel, S., Klompmaker, A.A., Mörs, T., Robins, C.M., and Kaim, A. 2019. A late Paleocene fauna from shallow-water chemosynthesis-based ecosystems, Spitsbergen, Svalbard. Acta Palaeontologica Polonica 64 (1): 101–141.

Hybertsen, F. and Kiel, S. 2018. A middle Eocene seep deposit with silicified fauna from the Humptulips Formation in western Washington State, USA. Acta Palaeontologica Polonica 63 (4): 751–768.

Kiel, S., Taviani, M. 2017. Chemosymbiotic bivalves from Miocene methane seep carbonates in Italy. Journal of Paleontology 91, 444–466.

Kiel, S. and Taviani, M. 2018. Chemosymbiotic bivalves from the late Pliocene Stirone River hydrocarbon seep complex in northern Italy. Acta Palaeontologica Polonica 63 (3): 557–568.

Kiel, S., Sami, M., and Taviani, M. 2018. A serpulid-Anodontia-dominated methane-seep deposit from the Miocene ofnorthern Italy. Acta Palaeontologica Polonica 63 (3): 569–577.

Krzysztof Hryniewicz
